# Bone marrow characterization in COPD: a multi-level network analysis

**DOI:** 10.1186/s12931-018-0824-x

**Published:** 2018-06-15

**Authors:** Nuria Toledo-Pons, Guillaume Noell, Andreas Jahn, Amanda Iglesias, Maria Antonia Duran, Julio Iglesias, Angel Rios, Sergio Scrimini, Rosa Faner, Orlando Gigirey, Alvar Agustí, Borja G. Cosío

**Affiliations:** 1 CIBER Enfermedades Respiratorias (CIBERES), Instituto de Salud Carlos III, Madrid, Spain; 2 Department of Respiratory Medicine, Hospital Universitari Son Espases-IdISBa, Valldemossa 79, 07010 Palma de Mallorca, Spain; 3 Institut d’investigacions Biomèdiques August Pi i Sunyer (IDIBAPS), Barcelona, Spain; 4 Department of Hematology, Hospital Universitari Son Espases-IdISBa, Mallorca, Spain; 5 Department of Immunology, Hospital Universitari Son Espases-IdISBa, Mallorca, Spain; 6 Department of Thoracic Surgery, Hospital Universitari Son Espases-IdISBa, Mallorca, Spain; 7 Department of Medicine, Universitat de Barcelona, Barcelona, Spain; 8 Respiratory Institute, Hospital Clinic, University of Barcelona, Barcelona, Spain

**Keywords:** COPD, Bone marrow, Eosinophil, Network

## Abstract

**Background:**

Bone marrow (BM) produces hematopoietic and progenitor cells that contribute to distant organ inflammation and repair. Chronic obstructive pulmonary disease (COPD) is characterized by defective lung repair. Yet, BM composition has not been previously characterized in COPD patients.

**Methods:**

In this prospective and controlled study, BM was obtained by sternum fine-needle aspiration in 35 COPD patients and 25 healthy controls (10 smokers and 15 never-smokers). BM cell count and immunophenotype were determined by microscopy and flow cytometry, respectively. Circulating inflammatory (C-reactive protein, IL-6, IL-8) and repair markers (HGF, IGF, TGF-β, VEGF) were quantified by ELISA. Results were integrated by multi-level network correlation analysis.

**Results:**

We found that: (1) there were no major significant pair wise differences between COPD patients and controls in the BM structural characteristics; (2) multi-level network analysis including patients and controls identifies a relation between immunity, repair and lung function not previously described, that remains in the COPD network but is absent in controls; and (3) this novel network identifies eosinophils as a potential mediator relating immunity and repair, particularly in patients with emphysema.

**Conclusions:**

Overall, these results suggest that BM is activated in COPD with impaired repair capacity in patients with more emphysema and/or higher circulating eosinophils.

**Electronic supplementary material:**

The online version of this article (10.1186/s12931-018-0824-x) contains supplementary material, which is available to authorized users.

## Background

The human bone marrow (BM) produces hematopoietic cells that contribute to the inflammatory response [1], as well as hematopoietic stem cells (HSC; CD34+) and endothelial progenitor cells (EPC; CD34+ CD133+ KDR+) that can migrate to distant tissues, such as the lungs, and modulate both the inflammatory response and tissue repair [2–5].

The role of some repair markers and growth factors have been studied in COPD patients or pre-clinical COPD models. It has been described that human mesenchymal stromal cells exert hepatocyte growth factor (HGF) dependent cytoprotective effects in a human relevant pre-clinical model of COPD [6]. Also, impaired vascular endothelial growth factor (VEGF) signalling has been associated with emphysema in animal models [7]. Recently, a possible novel role of insulin-like growth factor (IGF) has been described. IGF participate in the control of airway epithelial cell differentiation and the bronchiolar airway epithelial repair kinetics regulation after injury in various pulmonary diseases, such as asthma and COPD [8]. Finally, the transforming growth factor-beta (TGF-β) family regulates cell proliferation, differentiation, extracellular matrix synthesis, and apoptosis. Attenuation of TGF-β signalling leads to pulmonary emphysema in animal models [9] and has been shown to be decreased in the bronchiolar epithelium in patients with stable COPD compared with control smokers with normal lung function [10].

Chronic obstructive pulmonary disease (COPD) is characterized by both pulmonary and systemic inflammation, as well as by defective lung repair [11]. Indirect evidence supports the hypothesis that BM function in COPD is altered, as suggested by the reduced number of circulating-HSC and EPC in these patients [12], particularly in those with more severe airflow limitation [13] and low body mass index (BMI) values [14]. However, a recent small study did not identify significant differences in BM progenitor cell markers between 9 COPD patients and 9 age-matched, never-smoking controls [15]. Accordingly, in the present study we sought to use a holistic approach (network analysis [16]) to compare the characteristics of BM (and circulatory inflammation and repair markers) in a larger series of COPD patients and smoking and never-smoking controls with normal spirometry. Further, because patients with emphysema generally have severe airflow limitation and low BMI values [16], we hypothesized that differences would be magnified in these subgroup of patients. Finally, given that high circulating eosinophil levels (Eos), another BM-derived cell, seem to relate to clinical outcomes in COPD [17, 18], we also contrasted COPD patients with less or ≥ 300 circulating Eos/μL.

## Methods

### Study design and ethics

We included in this prospective and controlled study 35 clinically stable COPD patients defined by GOLD criteria [11] and 25 healthy controls (10 current smokers and 15 never smokers with normal spirometry) who required planned cardio-thoracic surgery for clinical reasons (COPD and smoker controls had indication for lung resection due to lung nodule or cancer by thoracic surgery and never-smoker controls had indication for valve replacement by cardiac surgery).

### Lung function

Forced spirometry, static lung volumes and carbon monoxide diffusing capacity (DLCO) were determined following international recommendations, using Mediterranean reference values [19, 20] in the pre-anaesthetic visit before surgery.

### Bone marrow harvesting and analysis

Sternum fine-needle (18G) BM aspiration (3–5 ml) was performed by an expert haematologist in the surgical theatre, once the patient was under general anaesthesia and before the sternotomy. Fresh BM smears were used for direct cell count (only in COPD patients). A 50 μl BM sample was diluted 1:4 in 1× PBS (pH 7,4; PAA) and analyzed using CELL-DYN Sapphire (Abbot, USA) to determine cell types distribution. HSC and EPC were isolated from fresh BM aspirates using a RosetteSep Kit (STEMCELL Technologies Inc., Canada) following manufacturer’s instructions to obtain a more representative sample. Cells were quantified by flow cytometry on a FACScan (Coulter Epics XL-MCL; Beckmann Coulter) by the presence of antigens CD34 (BD Pharmingen, NJ, USA), KDR (R&D Systems, Minneapolis, USA), CD133 (Miltenyi Biotec, Germany), c-kit, Ki67 (Abcam, Cambridge, UK). Gates were set to detect CD34+ cells and to evaluate co-expressions of CD34+ cells with CD 133, KDR, c-kit and Ki67. At least 20.000 events were acquired for each sample. Following previous publications [2–4], EPCs were also identified by FACS as CD34+ CD133+ KDR+ cells. The remaining BM sample were centrifuged at 1800 rpm/5 min/4 °C to separate cells from plasma and the supernatant was stored (100 μl aliquots) at − 80 °C until cytokine analysis.

### Circulating inflammatory and tissue repair markers

Circulating blood (10 ml) was collected in tubes with or without EDTA by venipuncture before anaesthesia. Total and differential leukocyte counts were determined automatically (Sysmex K-4500, Toa Medical Electronics Co Ltd., Kobe, Japan). Blood was allowed to clot and serum was separated by centrifugation at 3000 rpm for 15 min at 4 °C and stored at − 80 °C until analysis. The concentration of IL-6 (Labclinics, Barcelona, Spain), IL-8 (Invitrogen, California, USA), VEGF (Ebioscience), TGF-β (Ebioscience), IGF (R&D Systems) and HGF (Invitrogen) in plasma (and BM supernatant) were determined by ELISA. Assay sensitivities, as expressed by the manufacturer, were: 0.03 pg/ml for IL-6, < 100 fg/ml for IL-8, 7.9 pg/ml for VEGF, 8.6 pg/ml for TGF-β, 0.056 ng/mL for IGF and 20 pg/ml for HGF. C-reactive protein (CRP) concentration was determined by Nephelometry.

### Data and network analysis

Results are expressed as mean ± standard deviation, or median [interquartile range] values. ANOVA (or Kruskall-Wallis test) and unpaired t-test (or Mann-Whitney U-tests) were used to compare normally (and abnormally) distributed variables. Bivariate correlations were tested with Spearman test. Differences were considered statistically significant at 2-tailed *p* < 0.05 and the stronger of these Spearman relationships (threshold *p*-value < 0.01) between clinical and biological variables were plotted together as multi-level networks by representing variables as nodes and correlations between them as edges as previously described [21–23]. These correlation networks were constructed with custom R scripts and Cytoscape [24]. The multi-level network includes variables from multiple levels (or categories), including clinical, lung function, BM and circulating blood information. Each node in the network corresponds to a specific variable (see names) whose colour denotes the level to which it belongs (see legends). Nodes are linked by edges only if their Spearman correlation was statistically significant (*p* < 0.01), either positively (continuous lines) or negatively (dashed lines). The thickness of the edge is proportional to the correlation coefficient (Rho) (see legends). Grey shaded areas highlight 5 isolated modules (or sub-networks) that are named arbitrarily based on the assessment by the investigators of the type of variables included in each of them. In order to compare different biological effects, we built different networks comparing COPD and controls, COPD with DLCO< 60% or ≥ 60%, COPD with blood eosinophils< 300 or ≥ 300 cells/μL, and COPD with a smoking history< 45 or ≥ pack-years.

## Results

### Bivariate comparisons across groups

Table 1 presents the main demographic and clinical characteristics of the three groups studied. By design, smoking history and lung function values were different between them. BMI was higher in never smokers (*p* < 0.05). COPD patients had moderate-severe airflow limitation, gas trapping and reduced DLCO.

**Table 1:** Demographic and clinical characteristics (mean ± SD) of participants. Bolded italic text highlight variables with statistically significant differences (*p* ≤ 0.05)

	Never-smokers (*n* = 15)	Smokers (*n* = 10)	COPD (n = 35)	*P*-value
Age, years	62.50 ± 16.69	63.95 ± 10.36	66.35 ± 8.10	0.35
***BMI, kg/m*** ^***2***^	***33.00 ± 4.96***	***27.86 ± 4.36***	***27.05 ± 4.20***	***< 0.01***
***Pack years***	***0.00 ± 0.00***	***45.4 ± 22.82***	***51.0 ± 26.03***	***< 0.01***
***FEV1, % reference***	***105.61 ± 14.53***	***100.88 ± 14.01***	***64.26 ± 20.79***	***< 0.01***
***FEV1/FVC, %***	***79.29 ± 6.18***	***78.47 ± 5.29***	***53.84 ± 13.74***	***< 0.01***
***RV, % reference***	***112.8 ± 25.10***	***99.88 ± 23.69***	***172.41 ± 56.27***	***< 0.01***
***DLCO, % reference***	***91.21 ± 24.01***	***82.79 ± 13.90***	***60.79 ± 17.15***	***< 0.01***

*BMI* Body mass index, *FEV1* Forced expiratory volume in 1st second, *FVC* Forced vital capacity, *RV* Residual volume, *DLCO* Diffusing capacity of the lung for carbon monoxide

The percentages of cells expressing immune-phenotypic (HSC (CD34+) and EPC (CD34+ CD133+ KDR+)) or proliferation and differentiation markers (Ki67+, c-kit+) were similar pair wisely across groups (Table 2). Likewise, we did not find significant differences between groups in inflammatory or repair biomarkers, neither in BM or circulating blood, with the exception of increased CRP levels in COPD patients and elevated circulating neutrophils in smokers (Table 2).

**Table 2:** Pair wise comparison of BM and circulating inflammatory and repair markers (median [IQR] or mean ± SD) in the three groups studied. Bolded italic text highlights variables with statistically significant differences (*p* ≤ 0.05)

	Never-smokers (*n* = 15)	Smokers (*n* = 10)	COPD (*n* = 35)	ANOVA (or KW) *P* Value
BM CHARACTERIZATION				
Immunophenotype				
CD3%	5.00 [5.00–6.00]	5.50 [3.30–7.00]	6.00 [4.00–9.50]	0.59
CD5%	5.00 [5.00–6.00]	5.00 [3.00–7.00]	6.00 [4.30–9.80]	0.40
CD7%	7.00 [5.30–8.00]	6.00 [4.00–9.00]	6.00 [5.00–9.80]	0.78
CD10%	20.00 [13.00–26.00]	19.00 [15.00–28.50]	19.50 [15.80–25.80]	0.79
CD19 + %	3.00 [2.00–4.50]	3.00 [2.00–6.30]	3.00 [2.00–4.00]	0.57
CD19 + CD10 + %	2.00 [1.30–3.00]	0.50[0.50–2.50]	2.00 [1.00–2.00]	0.62
CD33%	76.00 [68.50–80.00]	68.50 [61.30–79.00]	71.50 [65.00–80.00]	0.47
CD13%	34.50 [30.00–39.50]	30.00 [20.00–40.00]	30.00 [25.00–33.00]	0.43
CD14%	2.00 [1.30–3.80]	3.00 [2.00–4.00]	3.00 [2.00–4.00]	0.31
CD15%	70.00 [57.50–75.00]	62.50 [56.30–73.80]	65.00 [60.00–70.00]	0.70
CD117%	2.00 [1.50–2.00]	2.00 [2.00–2.80]	2.00 [2.00–2.80]	0.40
CD34%	1.00 [1.00–2.00]	2.00 [1.00–2.80]	1.00 [1.00–2.00]	0.50
DR%	8.00 [4.50–11.00]	9.50 [7.30–10.00]	8.00 [7.503–10.00]	0.81
CD4%	3.00 [3.00–3.00]	3.00 [2.00–3.40]	3.50 [3.00–4.80]	0.24
CD8%	2.00 [2.00–3.00]	2.00 [1.30–4.00]	3.00 [2.00–4.00]	0.66
CD56%	1.00 [1.00–1.80]	1.00 [1.00–2.00]	1.00 [1.00–1.00]	0.83
sCD64%	3.00 [1.50–5.00]	3.00 [2.00–4.00]	3.00 [2.00–4.00]	0.88
Progenitor cell surface markers				
CD34				
%	7.38 [2.28–16.23]	13.44 [10.50–18.02]	14.50 [9.83–24.10]	0.07
MFI	164.60 [110.50–202.80]	118.03 [54.99–335.73]	92.65 [65.85–113.10]	0.09
CD 34 + c-kit+				
%	76.40 [67.00–85.40]	65.98 [57.88–79.71]	81.90 [66.80–84.70]	0.41
MFI	24.70 [19.60–39.65]	19.95 [15.85–70.73]	38.10 [24.80–55.40]	0.36
CD 34 + Ki67+				
%	69.40 [30.30–77.40]	83.30 [59.99–97.64]	96.80 [73.30–97.90]	0.72
MFI	31.80 [22.35–52.90]	28.00 [21.75–70.65]	50.30 [43.30–74.30]	0.32
CD34 + 133 + KDR+				
%	1.10 [0.00–3.05]	1.23 [0.18–1.80]	4.35 [1.58–7.73]	0.51
MFI	78.00 [37.93–127.15]	112.40 [74.48–134.73]	49.25 [40.10–120.60]	0.33
INFLAMMATORY AND REPAIR MARKERS				
BM Supernatant				
IL6 pg/ml	12.20 [1.30–23.00]	13.06 [1.65–27.39]	9.80 [7.60–24.90]	0.06
IL-8 pg/ml	6.50 [3.90–10.70]	7.10 [3.00–8.70]	3.60 [2.40–10-90]	0.48
HGF pg/ml	2854.07 [646.71–22,718.86]	1290.97 [909.37–9284.43]	1689.59 [692.43–2466.46]	0.63
Circulating blood				
Leucocytes ×10^^9^/L	7.67 ± 3.43	8.41 ± 2.45	7.41 ± 1.88	0.52
***Neutrophils %***	***55.07 ± 18.58*** ^*^	***71.98 ± 11.92***	***61.69 ± 11.85***	***0.02***
***Eos %***	***2.13 ± 1.77***	***1.46 ± 1.19***	***3.14 ± 1.65*** ^***†***^	***0.01***
***CRP mg/dl***	***1.62 ± 2.05***	***0.90 ± 2.19***	***5.65 ± 12.57*** ^***‡***^	***0.02***
IL-6 pg/ml	0.10 [0.10–0.10]	0.10 [0.00–0.10]	0.10 [0.1–0.20]	0.14
IL-8 pg/ml	1.40 [0.90–2.50]	2.80 [1.30–5.90]	2.30 [1.5–3.10]	0.82

*IL* Interleukin, *HCG* Hepatocyte growth factor, *MFI* Mean fluorescence intensity; ^*^P-value< 0.05 compared to never-smoking controls; ^***†***^P-value< 0.05 compared to smoking controls; ^***‡***^*P*-value< 0.05 compared to never-smoking and smoking controls

### Multi-level network analysis

#### All participants

**Fig. 1:**
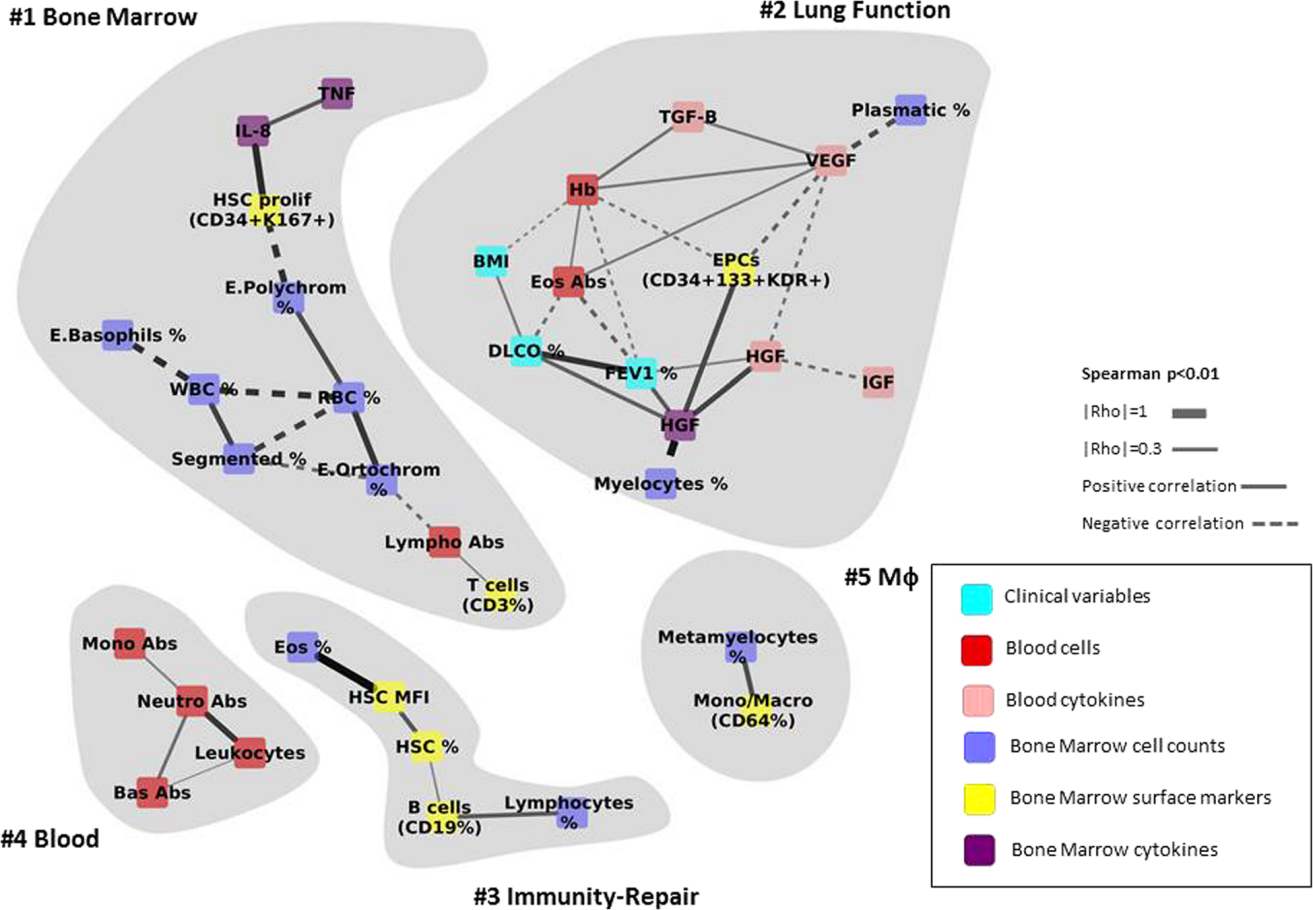
Multi-level correlation network that includes all participants in the study. For further explanations, see text. Abbreviations: Abs: absolute count; HSC: Hematopoietic stem cells; EPC: Endothelial progenitor cells; TNF: Tumour necrosis factor; TGF-ß: Transforming growth factor beta; VEGF: Vascular endothelial growth factor; IGF: Insulin-like growth factor; IL: Interleukin; RBC: Red blood cells; WBC: White blood cells; Lympho: Lymphocytes; Segmented: segmented neutrophils; E. Polychrom: Polychromatic erythroblast; E. Basophils: Basophils erythroblast; E. Ortochrom: Ortochromatic erythroblast; T cells: T lymphocytes; B cells: B lymphocytes; Plasmatic: Plasmatic cells; Hb: haemoglobin; Eos: Eosinophils; Mono/Macro: Monocyte/Macrophages; MFI: Mean fluorescence intensity; Mono: Monocyte; Neutro: Neutrophil; Bas: Basophil; Mφ: Macrophage; FEV1: forced expiratory volume in 1st second; DLCO: Diffusing capacity of the lung for carbon monoxide; BMI: body mass index

We first built a multi-level correlation network (Fig. 1) that included all participants (*n* = 60). The following observations are worth noting in Fig. 1: *(#1)* the *Bone Marrow module* has 11 nodes, all of them corresponding to BM measurements; *(#2)* the *Lung Function module* is the largest one (*n* = 13) and relates, as expected, FEV1, DLCO and BMI. Interestingly, it also includes several growth factors (HGF in BM as well as HGF, VEGF, IGF in peripheral blood), repair factors (TGF-β) and the percentatge of BM EPCs. Besides, circulating Eos relate inversely to both FEV1 and DLCO; *(#3)* the *Immunity-Repair module* relates the percentage of BM lymphocytes, B cells (CD19) and Eos with that of HSC (CD34); *(#4)* the *Blood module* includes a well defined sub-network of absolute counts of circulating cells, to some extend equivalent to those found in #1 (Bone Marrow), which included only BM markers; and, finally, *(#5)* the *Mϕ (Macrophage) module*, relates the levels of BM metamyelocytes and Monocytes/ Mϕ. All in all, these observations indicate the existence of a number of modules that can be “expected” physiologically (#1, #4-#5), but highlight that of some “unexpected” modules *(#2 Lung function and #3* the *Immunity-Repair module*) that are really multi-level and relate clinical, functional, BM and circulating markers of relevance in the context of the present analysis.

#### Comparison across groups: COPD vs. controls

**Fig. 2:**
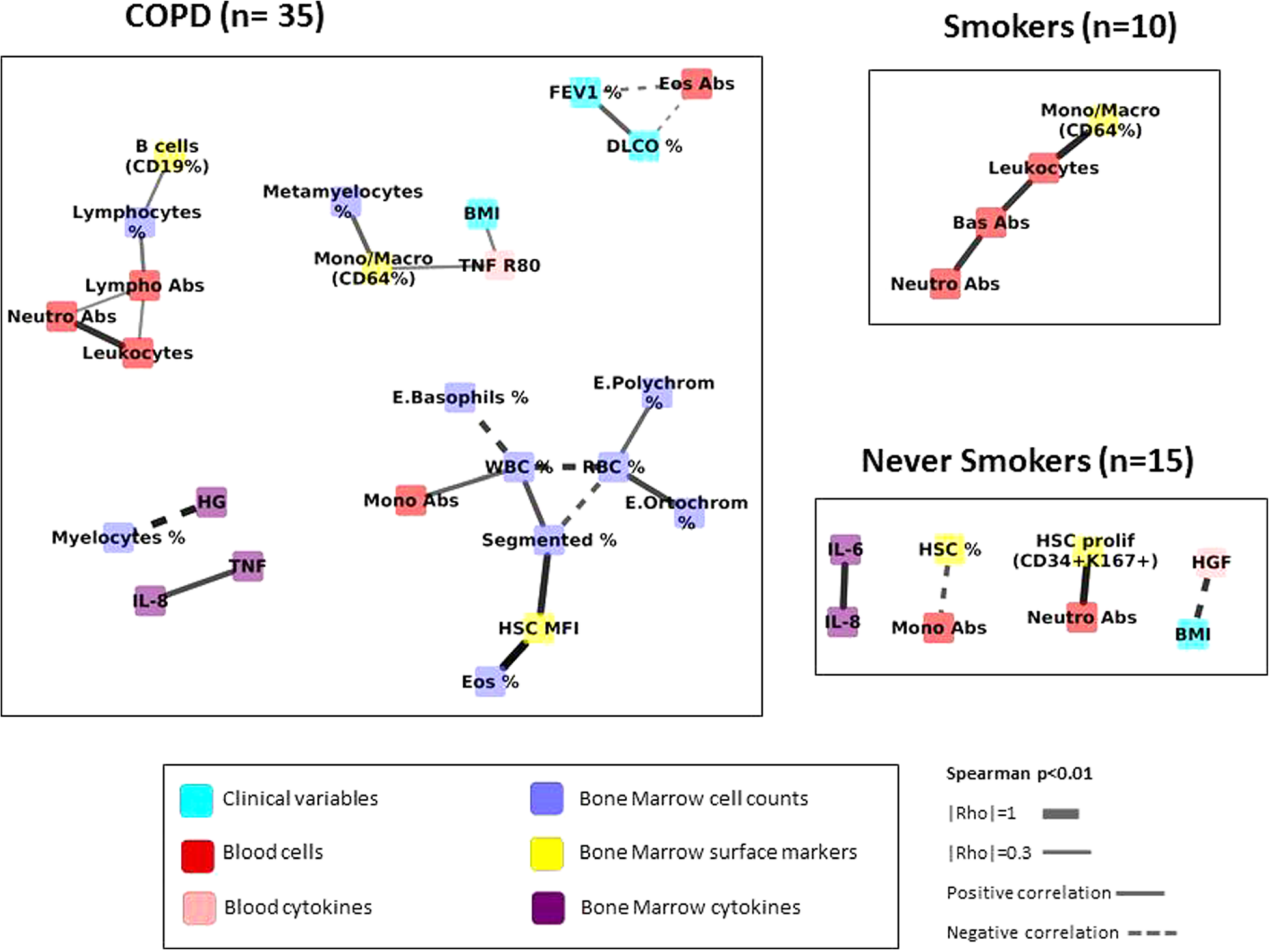
Multi-level correlation network calculated in never-smokers, smokers and COPD patients separately. For further explanations, see text. Abbreviations: Abs: absolute count; HSC: Hematopoietic stem cells, HSC prolif: Hematopoietic stem cells with a proliferative marker; TNF: Tumour necrosis factor; TNF R80: Tumour necrosis factor receptor p80; IL: Interleukin; RBC: Red blood cells; WBC: White blood cells; Lympho: Lymphocytes; Segmented: segmented neutrophils; E. Polychrom: Polychromatic erythroblast; E. Basophils: Basophils erythroblast; E. Ortochrom: Ortochromatic erythroblast; B cells: B lymphocytes, Eos: Eosinophils; Mono/Macro: Monocyte/Macrophages; MFI: Mean fluorescence intensity; Neutro: Neutrophil; Bas: Basophil; FEV1: forced expiratory volume in 1st second, DLCO: Diffusing capacity of the lung for carbon monoxide, BMI: body mass index

Figure 2 presents the correlation networks determined independently in COPD patients (*n* = 35), Smokers (*n* = 10) and Never-smokers (*n* = 15). It is important to note from the outset that the different number of individuals included in each network can modulate its structure, since the possibility of identifying significant correlations increases with sample size [25]. With this caveat in mind, Fig. 2 shows greater number of inter-connected nodes involved in the COPD network and that this network incorporates lung function variables, BM and blood markers of inflammation and repair that are not observed in controls (Fig. 2). To address the potential confounding effect of sample size differences, we increased the sample size of the control network by merging smokers and never smokers with normal spirometry (*n* = 25). Additional file 1: Figure S1 confirms that the COPD network continues to be more interconnected that that of merged controls, suggesting a higher level of system activation. Of note, FEV1, DLCO and circulating Eos are clearly related in COPD patients. Also, to assess the potential confounder effect of differential sample size on network density comparisons, we performed patients sub-sampling analysis in COPD or Controls. For each group, we averaged correlations coefficients and *p*-values of *n* = 1000 random subgroups of patients of n = 25, and observed that the difference in density is not due to differential sample size.

#### COPD phenotypes: Emphysema and circulating eosinophils

To get further insight into the relationship between emphysema, circulating Eos [26, 27] and BM response in COPD, as established a priori in the analysis plan, we first compared the results obtained in COPD patients with DLCO values higher or lower than 60% of reference, a well-established surrogate marker of emphysema [16]. As expected [16], lung function was worse in the latter patients (Additional file 3: Table S1). In patients with DLCO≥60% ref., bone marrow cellularity was enriched in white blood cells and segmented neutrophils (Additional file 3: Table S1). By contrast, the network was less structured in patients with DLCO< 60% ref., suggesting less network resilience [21] and impaired repair capacity in patients with emphysema (Additional file 2: Figure S2). Of note, circulating Eos were higher in these latter patients (Additional file 3: Table S1).

Secondly, we compared the network characteristics of COPD patients with less or ≥ 300 Eos/μL (Additional file 3: Table S2). The latter patients showed worse lung function and a reduced proportion of BM plasma cells and CD34+ c-kit+, as well as circulating neutrophils. The remaining BM and circulatory markers were similar in both groups. Additional file 4: Figure S3 shows that the multilevel correlation network was less well structured in patients with ≥300 circulating Eos/μL, suggesting again less resilience of the system [21].

## Discussion

The major novel findings of this study were that: [1] there were no major significant individual pair wise differences between COPD patients, smokers and never-smoker controls in the composition of immune-phenotypic, proliferation and differentiation, inflammatory or repair markers, neither in BM or circulating blood; [2] the novel multi-level correlation network approach used here identified (in all participants) a relation between immunity, repair and lung function not previously described; [3] network analysis also showed a greater number of interconnected nodes in COPD patients than in controls, with the presence of immunity and repair nodes not include in the network of controls; and [4] in COPD eosinophils appear as a possible central regulatory cell relating immunity and repair with lung function abnormalities and emphysema.

### Previous studies

Some previous reports have shown reduced BM-derived circulating progenitors (CD34+ cells) in patients with COPD, particularly in those with more severe airflow limitation, arterial hypoxemia and low-BMI [12, 14]. On the other, Broekman et al. recently described that the immune-phenotype, growth, differentiation and migration capacity of BM progenitor cells were not different between 9 COPD patients and 9 age-matched, never-smoking, controls [15]. The results of our pair wise analysis (Table 1) are in keeping with this latter report. However, our study extends all these previous studies by including a larger number of patients and controls and, specially, by using, for the first time, multilevel network analysis to get a holistic understanding of the system, both at the BM, circulating blood and physiologic levels.

### Interpretation of results

Despite that, by and large, we did not find significant pair wise differences between groups in the different immune-phenotypic, proliferation and differentiation, inflammatory or repair markers, neither in BM or circulating blood (Table 1), the use of multi-level correlation network analysis provided several observations of interests. Firstly, it identifies a module in which B-cells, progenitor cells (CD34+) and eosinophils interact in the whole population, as well as a second module that relates lung function parameters with immunity and repair nodes. Secondly, the network was more interconnected, hence more complex and developed, in COPD patients than in controls (Fig. 2 and Additional file 1: Figure S1), suggesting chronic system activation in COPD. The difference might, however, be due to the higher cumulated smoking history of COPD patients, as we have observed a denser network when comparing networks of patients with Pack-Years≥45 versus Pack-Years< 45 (Additional file 5: Figure S4).Thirdly, the COPD correlation network was more fragmented in those patients with emphysema (DLCO< 60% reference) or ≥ 300 circulating Eos/μL, indicating reduced system resilience [21] and potentially impaired repair capacity. This is consistent with the accepted pathogenesis of emphysema [28, 29] but highlights a novel patho-biological effect of eosinophils in the lung maintenance program. In fact, supporting this hypothesis and in keeping with previous studies [30, 31], we found that COPD patients with ≥300 Eos/μL had worse lung function and less BM HSCs (Table S2), suggesting deficient BM repair response. In support of this hypothesis is the recent observation in the COPDgene cohort of a relation between biomarkers of extracellular matrix turnover, emphysema and eosinophilic-bronchitis in patients with COPD [27]. Moreover, other studies have found involvement of eosinophils in colonic inflammation and repair [26]. The finding of eosinophils as cells that are present in almost all the networks being one of the most connected hubs in each network and also the different number of compartments in which it was involved (as clinical variables, blood cytokines, bone marrow surface markers) made us to consider the role of eosinophil as a possible regulatory cell.

Finally, we did not find differences in the bivariate analysis between different growth factors but the network analysis showed relationships among HGF, VEGF, TGF, EPCs, eosinophils and lung function parameters. It has been recently described that human mesenchymal stromal cells exert HGF dependent cytoprotective effects in a human relevant pre-clinical model of COPD [6], that is consistent with the role of HGF in the network connected with DLCO, FEV1 and EPC. Moreover, impaired VEGF signaling has been associated with emphysema in animal models [7] and our network shows an inverse correlation with EPC which could be explained as a consequence of the impaired repair mechanisms in these patients.

### Strengths and limitations

The use, for the first time, of network analysis to understand the BM system in a large cohort of COPD patients and controls is a clear strength of our study. It also has, however, some potential limitations that deserve comment. First, because COPD patients (and controls) required cardio-thoracic surgery for clinical reasons (with different indication for cases and controls), results may not be automatically generalised. Second, as discussed above, we studied a higher number of COPD patients than controls, and this may influence the structure of correlation networks [25]. To address this, we increased the number of controls by merging smokers and never smokers, and yet found similar results. However, we still could not correct for the effect of active smoking due to the sample size. Third, network density is also directly proportional to the threshold chosen for statistical significance, the lower the *p* value, the higher network density. To get an appropriate signal to noise balance, as others have done before [16, 21], we established an arbitrary p threshold value of less than 0.01, so weaker relationships were not included in the network. Fourth, although the data point to eosinophil as a possible mediator of the immune and repair response, especially in patients with emphysema, the authors cannot exclude the presence of other co-mediators and further studies are needed to demonstrate the role of these cells in the repair process of the lung. In keeping with this, due to the sample size we could not explore the effects of inhaled corticosteroids, that some of these patients were receiving, on the BM characteristics. Finally, we studied only one source of stem cells, namely the BM, while there are other organs also able to produce these cells, such as the spleen or even the lung themselves [32].

## Conclusions

These results suggest that BM seems to be chronically activated in COPD, despite there are not differences in the baseline structure and function between COPD and controls. Moreover, the resilience and repair capacity of the system can be impaired in patients with more emphysema and/or higher circulating Eos.

## Additional files

Additional file 1: **Figure S1.** Multi-level correlation network of COPD patients and merged controls (smokers and non-smokers with normal spirometry). For further explanations, see text. Abbreviations: Abs: absolute count; HSC: Hematopoietic stem cells, TNF R80: Tumour necrosis factor receptor p80; HGF: Hepatocyte growth factor; VEGF: Vascular endothelial growth factor, IL: Interleukin; RBC: Red blood cells; WBC: White blood cells; Lympho: Lymphocytes; Segmented: segmented neutrophils; E. Polychrom: Polychromatic erythroblast; E. Basophils: Basophils erythroblast; E. Ortochrom: Ortochromatic erythroblast; B cells: B lymphocytes; Hb: haemoglobin, Eos: Eosinophils; Mono/Macro: Monocyte/Macrophages; MFI: Mean fluorescence intensity; Neutro: Neutrophil; FEV1: forced expiratory volume in 1st second; DLCO: Diffusing capacity of the lung for carbon monoxide, BMI: body mass index. (JPG 51 kb)

Additional file 2: **Figure S2.** Multi-level correlation network of COPD patients with DLCO ≥60% or <60%. For further explanations, see text. Abbreviations: Abs: absolute count; HSC: Hematopoietic stem cells; EPC: Endothelial progenitor cells; TNF R80: Tumour necrosis factor receptor p80; TGF-ß: Transforming growth factor beta; HGF: Hepatocyte growth factor; VEGF: Vascular endothelial growth factor; IGF: Insulin-like growth factor; RBC: Red blood cells; Lympho: Lymphocytes; Segmented: segmented neutrophils; E. Polychrom: Polychromatic erythroblast; E. Ortochrom: Ortochromatic erythroblast; B cells: B lymphocytes; Plasmatic: Plasmatic cells; Hb: haemoglobin; Eos: Eosinophils; Mono/Macro: Monocyte/Macrophages; MFI: Mean fluorescence intensity; Mono: Monocyte; Neutro: Neutrophil; FEV1: forced expiratory volume in 1st second; DLCO: Diffusing capacity of the lung for carbon monoxide; BMI: body mass index. (JPG 60 kb)

Additional file 3: **Table S1.** Bone marrow characterization, inflammatory and repair markers (mean±SD or median [IQR]) in COPD patients with DLCO higher or lower than 60% of reference. Bolded italic text highlight variables with statistically significant differences ( p≤0.05). **Table S2.** Bone marrow characterization, inflammatory and repair markers (mean±SD or median [IQR]) in COPD patients with peripheral blood eosinophil counts <300 or ≥300/μL. Bolded italic text highlight variables with statistically significant differences (p≤0.05). (DOCX 42 kb)

Additional file 4: **Figure S3.** Multi-level correlation network of COPD patients with peripheral blood eosinophil counts <300/μL or ≥300/μL. For further explanations, see text. Abbreviations: Abs: absolute count; HSC: Hematopoietic stem cells; EPC: Endothelial progenitor cells; TNF R80: Tumour necrosis factor receptor p80; VEGF: Vascular endothelial growth factor; RBC: Red blood cells; WBC: White blood cells; Lympho: Lymphocytes; Segmented: segmented neutrophils; E. Ortochrom: Ortochromatic erythroblast; T cells: T lymphocytes; Plasmatic: Plasmatic cells; Mono/Macro: Monocyte/Macrophages; Mono: Monocyte; Neutro: Neutrophil; FEV1: forced expiratory volume in 1st second; DLCO: Diffusing capacity of the lung for carbon monoxide; BMI: body mass index. (JPG 53 kb)

Additional file 5: **Figure S4.** Multi-level correlation network of COPD patients with smoking history <45 pack-years or ≥45 pack-years. For further explanations, see text. Abbreviations: Abs: absolute count; HSC: Hematopoietic stem cells; TNF: Tumour necrosis factor; VEGF: Vascular endothelial growth factor; HGF: Hepatocyte growth factor; IL: Interleukin; RBC: Red blood cells; WBC: White blood cells; Lympho: Lymphocytes; B cells: B lymphocytes; T cells: T lymphocytes; Mono: Monocyte; Neutro: Neutrophil; Segmented: segmented neutrophils; Eos: Eosinophils; E. Ortochrom: Ortochromatic erythroblast; E. Polychrom: Polychromatic erythroblast; FEV1: forced expiratory volume in 1szt second; DLCO: Diffusing capacity of the lung for carbon monoxide; BMI: body mass index. (JPG 59 kb)

## Abbreviations

BM: Bone marrow
BMI: Body mass index
COPD: Chronic obstructive pulmonary disease
CRP: C-reactive protein
DLCO: Carbon monoxide diffusing capacity
ELISA: Enzyme-Linked Immunosorbent Assay
Eos: Eosinophil
EPC: Endothelial progenitor cells
FEV1: Forced expiratory volume in the first second
HGF: Hepatocyte growth factor
HSC: Hematopoietic stem cells
IGF: Insulin-like growth factor
IL: Interleukin
Mφ: Macrophage
TGF-β: Transforming growth factor beta
VEGF: Vascular endothelial growth factor
