# Supplementary figures and images for: Bone marrow characterization in COPD: a multi-level network analysis
Source: Respir Res. 2018 Jun 15;19:118. doi: 10.1186/s12931-018-0824-x (PMC6003122; doi:10.1186/s12931-018-0824-x)

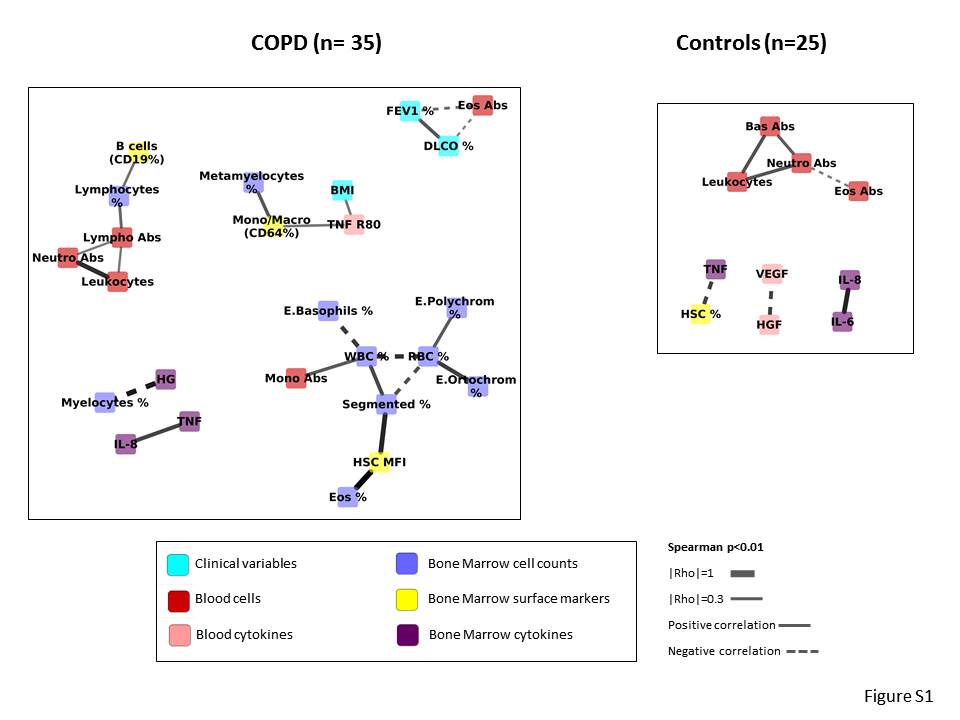

Supplement: Supplementary file 1 — Figure S1. Multi-level correlation network of COPD patients and merged controls (smokers and non-smokers with normal spirometry). For further explanations, see text. Abbreviations: Abs: absolute count; HSC: Hematopoietic stem cells, TNF R80: Tumour necrosis factor receptor p80; HGF: Hepatocyte growth factor; VEGF: Vascular endothelial growth factor, IL: Interleukin; RBC: Red blood cells; WBC: White blood cells; Lympho: Lymphocytes; Segmented: segmented neutrophils; E. Polychrom: Polychromatic erythroblast; E. Basophils: Basophils erythroblast; E. Ortochrom: Ortochromatic erythroblast; B cells: B lymphocytes; Hb: haemoglobin, Eos: Eosinophils; Mono/Macro: Monocyte/Macrophages; MFI: Mean fluorescence intensity; Neutro: Neutrophil; FEV1: forced expiratory volume in 1st second; DLCO: Diffusing capacity of the lung for carbon monoxide, BMI: body mass index. (JPG 51 kb) [file 12931_2018_824_MOESM1_ESM.jpg]

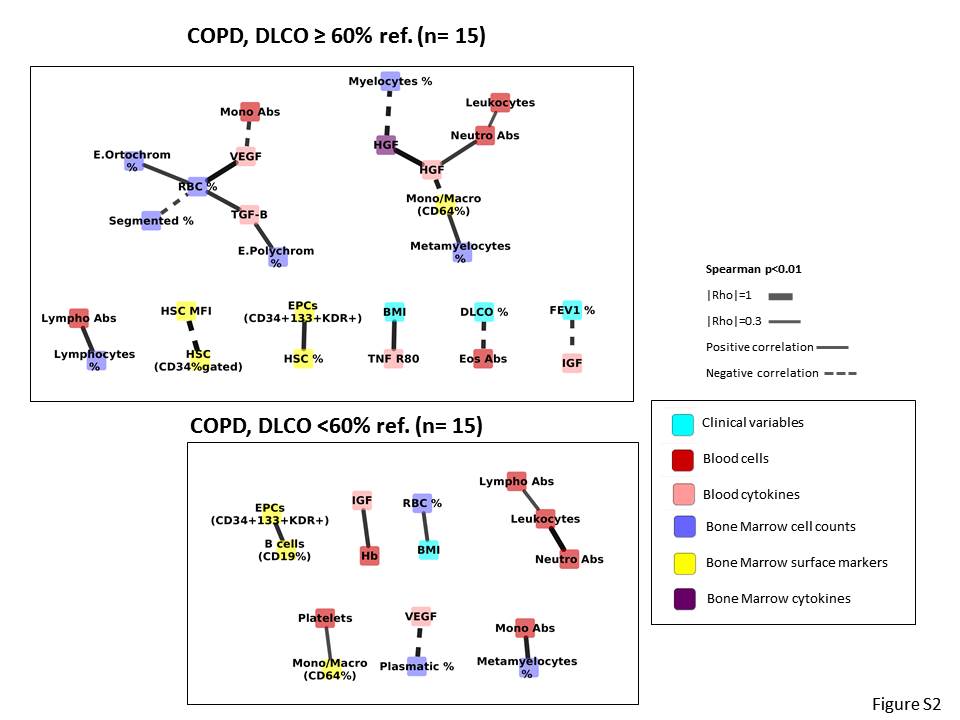

Supplement: Supplementary file 2 — Figure S2. Multi-level correlation network of COPD patients with DLCO ≥60% or <60%. For further explanations, see text. Abbreviations: Abs: absolute count; HSC: Hematopoietic stem cells; EPC: Endothelial progenitor cells; TNF R80: Tumour necrosis factor receptor p80; TGF-ß: Transforming growth factor beta; HGF: Hepatocyte growth factor; VEGF: Vascular endothelial growth factor; IGF: Insulin-like growth factor; RBC: Red blood cells; Lympho: Lymphocytes; Segmented: segmented neutrophils; E. Polychrom: Polychromatic erythroblast; E. Ortochrom: Ortochromatic erythroblast; B cells: B lymphocytes; Plasmatic: Plasmatic cells; Hb: haemoglobin; Eos: Eosinophils; Mono/Macro: Monocyte/Macrophages; MFI: Mean fluorescence intensity; Mono: Monocyte; Neutro: Neutrophil; FEV1: forced expiratory volume in 1st second; DLCO: Diffusing capacity of the lung for carbon monoxide; BMI: body mass index. (JPG 60 kb) [file 12931_2018_824_MOESM2_ESM.jpg]

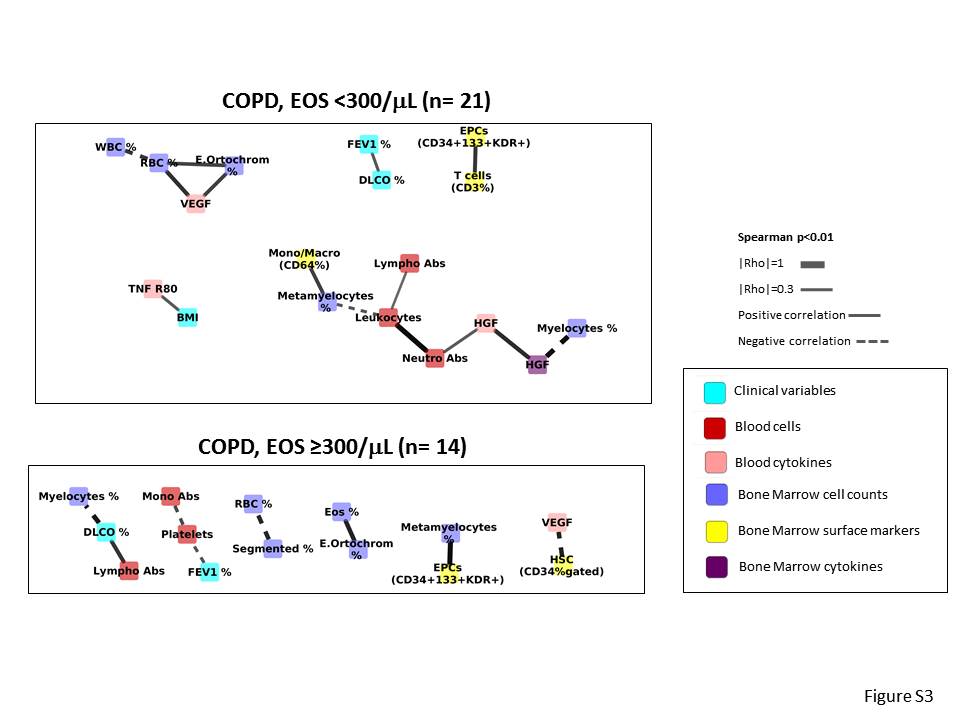

Supplement: Supplementary file 4 — Figure S3. Multi-level correlation network of COPD patients with peripheral blood eosinophil counts <300/μL or ≥300/μL. For further explanations, see text. Abbreviations: Abs: absolute count; HSC: Hematopoietic stem cells; EPC: Endothelial progenitor cells; TNF R80: Tumour necrosis factor receptor p80; VEGF: Vascular endothelial growth factor; RBC: Red blood cells; WBC: White blood cells; Lympho: Lymphocytes; Segmented: segmented neutrophils; E. Ortochrom: Ortochromatic erythroblast; T cells: T lymphocytes; Plasmatic: Plasmatic cells; Mono/Macro: Monocyte/Macrophages; Mono: Monocyte; Neutro: Neutrophil; FEV1: forced expiratory volume in 1st second; DLCO: Diffusing capacity of the lung for carbon monoxide; BMI: body mass index. (JPG 53 kb) [file 12931_2018_824_MOESM4_ESM.jpg]

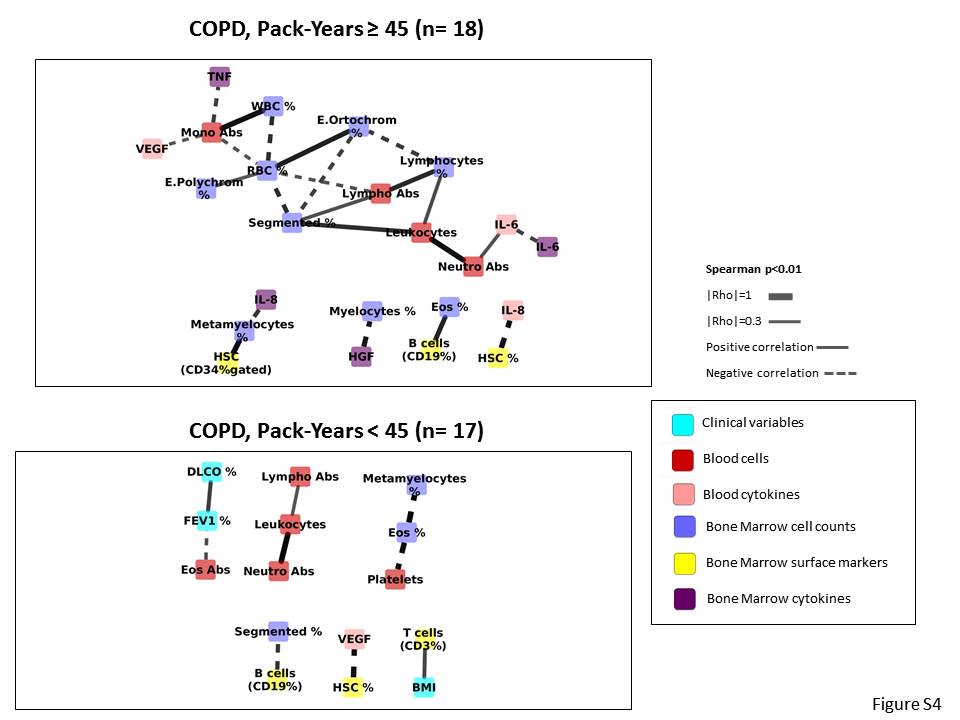

Supplement: Supplementary file 5 — Figure S4. Multi-level correlation network of COPD patients with smoking history <45 pack-years or ≥45 pack-years. For further explanations, see text. Abbreviations: Abs: absolute count; HSC: Hematopoietic stem cells; TNF: Tumour necrosis factor; VEGF: Vascular endothelial growth factor; HGF: Hepatocyte growth factor; IL: Interleukin; RBC: Red blood cells; WBC: White blood cells; Lympho: Lymphocytes; B cells: B lymphocytes; T cells: T lymphocytes; Mono: Monocyte; Neutro: Neutrophil; Segmented: segmented neutrophils; Eos: Eosinophils; E. Ortochrom: Ortochromatic erythroblast; E. Polychrom: Polychromatic erythroblast; FEV1: forced expiratory volume in 1szt second; DLCO: Diffusing capacity of the lung for carbon monoxide; BMI: body mass index. (JPG 59 kb) [file 12931_2018_824_MOESM5_ESM.jpg]
