# Supplementary material for: Bone marrow characterization in COPD: a multi-level network analysis
Source: Respir Res. 2018 Jun 15;19:118. doi: 10.1186/s12931-018-0824-x (PMC6003122; doi:10.1186/s12931-018-0824-x)
Supplement: Supplementary file 3 — Table S1. Bone marrow characterization, inflammatory and repair markers (mean±SD or median [IQR]) in COPD patients with DLCO higher or lower than 60% of reference. Bolded italic text highlight variables with statistically significant differences ( p≤0.05). Table S2. Bone marrow characterization, inflammatory and repair markers (mean±SD or median [IQR]) in COPD patients with peripheral blood eosinophil counts <300 or ≥300/μL. Bolded italic text highlight variables with statistically significant differences (p≤0.05). (DOCX 42 kb) [file 12931_2018_824_MOESM3_ESM.docx]

June 12, 2018

**On-line supplement**

**BONE MARROW CHARACTERIZATION IN COPD:**

**A MULTI-LEVEL NETWORK ANALYSIS**

Nuria Toledo-Pons^1,2^, Guillaume Noell^1,3^, Andreas Jahn^2^, Amanda Iglesias^1,2^,

Maria Antonia Duran^4^, Julio Iglesias^5^, Angel Rios^2^, Sergio Scrimini^2^, Rosa Faner^1,3^, Orlando Gigirey^6^, Alvar Agustí^1,3,7,8^, Borja G Cosío^1,2^.

**On-line supplement Tables**: 2; **Figures**: 4.

**Table S1.** Bone marrow characterization, inflammatory and repair markers (mean±SD or median [IQR]) in COPD patients with DLCO higher or lower than 60% of reference. ***Bolded*** ***italic*** text highlight variables with statistically significant differences ( p≤0.05).

|  | **DLCO≥60% ref (n=15)** | **DLCO<60% ref (n=15)** | **P Value** |
| --- | --- | --- | --- |
| **DEMOGRAPHICS** | | | |
| Age (years) | 66.45±8.53 | 64.50±6.73 | 0.42 |
| IMC (kg/m2) | 27.79±3.59 | 26.47±4.97 | 0.33 |
| Pack years | 44.06±25.07 | 54.56±26.05 | 0.23 |
| ***FEV1 % predicted*** | ***72.18±16.23*** | ***50.95±18.16*** | ***<0.01*** |
| ***FEV1/FVC %*** | ***60.45±10.20*** | ***46.00±13.82*** | ***<0.01*** |
| RV % predicted | 155.75±49.90 | 189.35±61.40 | 0.08 |
| ***DLCO % predicted*** | ***73.41±11.15*** | ***46.90±10.46*** | ***<0.01*** |
| **BONE MARROW CHARACTERIZATION** | | | |
| **Cell count** |  |  |  |
| Red blood cells % | 39.00 [28.00-45.00] | 26.50 [23.50-39.00] | 0.09 |
| Proerythroblast % | 1.00 [1.00-1.00] | 1.00 [1.00-1.00] | 0.66 |
| Basophil erythroblast % | 1.50 [1.00-2.75] | 1.00 [1.00-1.00] | 0.28 |
| Polychromatic erythroblast % | 16.00 [9.00-21.50] | 13.00 [10.00-16.00] | 0.46 |
| Orthochromatic erythroblast % | 19.00 [15.50-23.00] | 12.00 [11.00-20.00] | 0.09 |
| Lymphocytes % | 9.00 [6.00-10.50] | 7.00 [4.00-12.00] | 0.69 |
| Plasmatic cells % | 2.00 [1.00-3.00] | 1.00 [1.00-2.00] | 0.27 |
| ***White blood cells %*** | ***48.00 [44.00-57.50]*** | ***61.00 [55.00-66.75]*** | ***0.03*** |
| Myeloblasts % | 1.00 [0.75-1.00] | 1.00 [1.00-1.75] | 0.35 |
| Myelocytes % | 15.50 [13.25-17.00] | 17.50 [16.00-19.75] | 0.14 |
| Metamyelocytes% | 18.00 [16.50-24.50] | 22.00 [19.25-23.50] | 0.21 |
| ***Segmented Neutrophils %*** | ***13.00 [11.00-16.50]*** | ***18.00 [14.00-20.00]*** | ***0.04*** |
| Eosinophils % | 5.00 [4.00-8.50] | 3.50 [2.00-5.00] | 0.08 |
| **Immunophenotype** |  |  |  |
| CD3 % | 5.50 [4.25-9.50] | 7.00 [4.00-10.00] | 0.49 |
| CD5 % | 5.50 [5.00-9.50] | 7.00 [4.00-10.00] | 0.46 |
| CD7 % | 6.50 [5.00-8.75] | 7.00 [5.00-11.00] | 0.46 |
| CD10 % | 18.50 [15.00-25.00] | 20.00 [18.00-30.00] | 0.52 |
| CD19 % | 3.00 [2.00-4.00] | 2.00 [1.00-3.00] | 0.30 |
| CD19+CD10 % | 1.00 [0.50-2.00] | 0.50 [0.50-1.00] | 0.37 |
| CD33 % | 72.00 [64.25-80.00] | 70.00 [65.00-76.00] | 0.89 |
| CD13 % | 30.50 [30.00-33.75] | 30.00 [20.00-33.00] | 0.37 |
| CD14 % | 3.00 [2.00-4.50] | 3.00 [3.00-4.00] | 0.76 |
| CD15 % | 61.00 [55.75-69.50] | 65.00 [60.00-75.00] | 0.40 |
| CD117 % | 2.00 [2.00-2.75] | 2.00 [1.00-3.00] | 0.46 |
| CD34 % | 1.00 [1.00-2.00] | 1.00 [1.00-2.00] | 0.90 |
| DR % | 8.00 [7.00-8.00] | 9.00 [8.00-10.00] | 0.11 |
| CD4 % | 3.00 [2.25-4.75] | 4.00 [3.00-6.00] | 0.40 |
| CD8 % | 3.00 [2.00-4.00] | 3.00 [2.00-4.00] | 0.95 |
| CD56 % | 1.00 [1.00-1.00] | 1.00 [1.00-2.00] | 0.35 |
| CD64 % | 3.00 [2.00-4.50] | 3.00 [3.00-4.00] | 0.76 |
| **Progenitor cell surface markers** | | | |
| CD34 |  |  |  |
| % | 5.60 [2.03-11.95] | 11.10 [1.70-18.00] | 0.32 |
| MFI | 185.50 [154.30-227.50] | 116.40 [102.40-191.60] | 0.18 |
| CD 34+ckit |  |  |  |
| % | 5.60 [2.03-11.95] | 11.10 [1.70-18.00] | 0.32 |
| MFI | 185.50 [154.30-227.50] | 116.40 [102.40-191.60] | 0.18 |
| CD 34+Ki67+ % |  |  |  |
| % | 69.60 [12.33-82.70] | 69.40 [30.50-79.75] | 0.79 |
| MFI | 48.15 [29.40-59.78] | 25.00 [20.80-42.95] | 0.18 |
| CD34+133+KDR+ % |  |  |  |
| % | 0.70 [0.00-2.80] | 3.20 [0.52-12.30] | 0.09 |
| MFI | 115.10 [74.75-157.15] | 41.30 [24.80-124.10] | 0.15 |
| **INFLAMMATORY AND REPAIR MARKERS** | | | |
| **BM Supernatant** |  |  |  |
| IL6 pg/ml | 14.48 [9.14-25.73] | 8.42 [7.30-21.22] | 0.35 |
| IL-8 pg/ml | 5.33 [3.56-13.16] | 2.52 [2.23-12.25] | 0.41 |
| HGF pg/ml | 2.358.40 [622.79-4.576.24] | 1.603.61 [931.35-1.858.95] | 0.49 |
| **Circulating blood** |  |  |  |
| Leucocytes x109/L | 7.85±2.20 | 7.45±1.44 | 0.56 |
| Neutrophils % | 64.01±7.98 | 61.97±5.24 | 0.42 |
| ***Eosinophils x10^9/L*** | ***0.19±0.12*** | ***0.28±0.13*** | ***0.05*** |
| ***Eosinophils %*** | ***2.49±0.95*** | ***3.75±1.96*** | ***0.04*** |
| CRP mg/dl | 2.00 [0.00-8.50] | 2.00 [0.25-10.25] | 0.99 |
| IL-6 pg/ml | 0.10 [0.09-0.20] | 0.13 [0.10-0.22] | 0.64 |
| IL-8 pg/ml | 1.99 [1.16-3.24] | 2.34 [1.81-2.82] | 0.53 |
| HGF pg/ml | 567.72 [468.63-807.10] | 614.69 [435.79-748.89] | 0.89 |
| IGF ng/ml | 72.90 [46.35-88.75] | 52.20 [46.40-75.60] | 0.31 |
| TGF-β pg/ml | 12.510.18 [9.336.71-18.938.31] | 11.495 [9.735.00-23.768.76] | 0.77 |
| VEGF pg/ml | 150.19 [110.31-344.95] | 137.24 [54.78-195.07] | 0.31 |

*Five patients are missing due to difficulties to perform DLCO maneuvers.

IL: interleukin; HGF: hepatocyte growth factor; IGF: insulin-like growth factor; TGF-β: transforming growth factor β, VEGF: vascular endothelial growth factor.

**Table S2.** Bone marrow characterization, inflammatory and repair markers (mean±SD or median [IQR]) in COPD patients with peripheral blood eosinophil counts <300 or ≥300/µL. ***Bolded*** ***italic*** text highlight variables with statistically significant differences (p≤0.05).

|  | **COPD <300 Eos**/µL  **(n=14)** | **COPD ≥300 Eos**/µL  **(n=21)** | **P Value** |
| --- | --- | --- | --- |
| **DEMOGRAPHICS** | | | |
| Age (years) | 69.75±7.89 | 64.71±7.15 | 0.13 |
| IMC (kg/m2) | 28.41±3.45 | 25.45±5.29 | 0.06 |
| Pack years | 45.00±23.00 | 60.00±29.00 | 0.19 |
| ***FEV1 % predicted*** | ***64.95±21.31*** | ***48.77±15.32*** | ***0.02*** |
| ***FEV1/FVC %*** | ***54.57±12.87*** | ***44.69±14.86*** | ***0.04*** |
| RV % predicted | 174.75±51.69 | 201.18±50.33 | 0.20 |
| ***DLCO % predicted*** | ***64.16±16.52*** | ***49.30±13.74*** | ***0.02*** |
| **BONE MARROW CHARACTERIZATION** | | | |
| **Cell count** |  |  |  |
| Red blood cells % | 37.00 [25.30-43.50] | 29.50 [25.00-36.50] | 0.19 |
| Proerythroblast % | 1.00 [1.00-1.00] | 1.00 [1.00-1.00] | 0.86 |
| Basophil erythroblast % | 1.00 [1.00-2.00] | 1.00 [1.00-1.50] | 0.72 |
| Polychromatic erythroblast % | 14.50 [8.50-22.30] | 14.00 [10.00-15.30] | 0.55 |
| Orthochromatic erythroblast % | 18.00 [12.00-23.00] | 15.50 [11.30-20.00] | 0.45 |
| Lymfocites % | 7.50 [4.00-10.30] | 10.50 [6.80-12.80] | 0.06 |
| ***Plasmatic cells %*** | ***2.00 [1.00-3.00]*** | ***1.00 [1.00-1.00]*** | ***0.03*** |
| White blood cells % | 54.50 [47.50-59.30] | 60.00 [55.00-64.00] | 0.15 |
| Myeloblasts % | 1.00 [1.00-1.00] | 1.50 [1.00-2.00] | 0.16 |
| Myelocytes % | 16.00 [13.80-17.80] | 17.00 [15.00-19.00] | 0.71 |
| Metamyelocytes % | 19.00 [17.00-25.00] | 22.00 [17.00-25.50] | 0.73 |
| Segmented Neutrophils % | 14.00 [12.00-17.00] | 18.00 [13.50-19.50] | 0.12 |
| Eosinophils % | 4.00 [2.50-5.00] | 5.00 [4.30-7.80] | 0.14 |
| **Immunophenotype** |  |  |  |
| CD3 % | 6.50 [4.25-10.00] | 5.50 [4.00-7.00] | 0.40 |
| CD5 % | 6.50 [5.00-10.00] | 5.59 [4.00-8.50] | 0.45 |
| CD7 % | 7.50 [5.00-10.00] | 6.00 [5.00-7.50] | 0.40 |
| CD10 % | 18.50 [15.00-24.25] | 23.00 [18.25-30.00] | 0.16 |
| CD19 % | 2.00 [2.00-3.75] | 3.00 [2.25-4.00] | 0.35 |
| CD19+CD10 % | 1.00 [0.50-2.00] | 0.75 [0.50-2.00] | 0.33 |
| CD33 % | 74.50 [66.25-80.00] | 70.00 [65.00-78.75] | 0.66 |
| CD13 % | 31.50 [26.25-33.00] | 30.00 [20.00-38.00] | 0.39 |
| CD14 % | 3.00 [2.00-3.75] | 3.00 [3.00-4.00] | 0.54 |
| CD15 % | 66.00 [58.50-73.75] | 60.00 [60.00-70.00] | 0.60 |
| CD117 % | 2.00 [2.00-2.75] | 2.00 [1.00-2.75] | 0.51 |
| CD34 % | 2.00 [1.00-2.00] | 1.00 [1.00-2.00] | 0.24 |
| DR % | 8.00 [7.25-10.00] | 8.00[7.25-10.00] | 0.87 |
| CD4 % | 4.00 [2.25-5.00] | 3.00 [3.00-4.00] | 0.42 |
| CD8 % | 3.00 [2.00-4.00] | 2.50 [2.00-3.00] | 0.28 |
| CD56 % | 1.00 [1.00-1.00] | 1.00 [1.00-1.00] | 0.88 |
| CD64 % | 300 [2.00-3.75] | 3.00 [3.00-4.00] | 0.61 |
| **Progenitor cell surface markers** | | | |
| CD34+ |  |  |  |
| % | 9.65 [2.88-16.70] | 5.15 [1.53-16.35] | 0.39 |
| MFI | 173.80 [108.48-200.40] | 124.00 [96.20-216.20] | 1.00 |
| CD 34+ckit+ |  |  |  |
| % | 81.80 [74.75-86.65] | 69.10 [55.95-77.60] | 0.13 |
| ***MFI*** | ***36.45 [21.93-45.93]*** | ***18.70 [16.15-28.45]*** | ***0.03*** |
| CD 34+Ki67+ |  |  |  |
| % | 69.60 [59.88-83.88] | 47.50 [7.15-75.20] | 0.22 |
| MFI | 28.40 [22.28-48.73] | 33.30 [25.20-61.25] | 0.52 |
| CD34+133+KDR+ |  |  |  |
| % | 1.62 [0.26-3.40] | 0.70 [0.00-5.23] | 0.71 |
| MFI | 74.90 [26.30-119.60] | 112.20 [58.45-153.15] | 0.30 |
| **INFLAMMATORY AND REPAIR MARKERS** | | | |
| **BM Supernatant** |  |  |  |
| IL6 pg/ml | 9.37 [8.42-20.12] | 14.47 [7.94-26.17] | 0.54 |
| IL-8 pg/ml | 3.70 [2.32-7.80] | 4.77 [2.33-14.36] | 0.74 |
| HGF pg/ml | 1.809.14 [630.75-3.903.29] | 957.25 [656.60-1.925.63] | 0.42 |
| **Circulating blood** |  |  |  |
| Leucocytes x109/L | 7.23±1.87 | 7.69±1.94 | 0.485 |
| ***Neutrophils %*** | ***64.84 ±7.13*** | ***56.97±15.79*** | ***0.05*** |
| ***Eos %*** | ***2.23±0.79*** | ***4.49±1.71*** | ***<0.01*** |
| CRP mg/dl | 8.76±7.94 | 11.07±22.59 | 0.34 |
| IL-6 pg/ml | 0.12 [0.9-0.21] | 0.11 [0.06-0.18] | 0.75 |
| IL-8 pg/ml | 2.27 [1.42-3.10] | 2.43 [1.46-4.07] | 0.56 |
| HGF pg/ml | 633.15 [464.24-872.44] | 593.33 [457.84-781.33] | 0.62 |
| IGF ng/ml | 53.65 [43.70-82.40] | 70.20 [46.55-78.45] | 0.59 |
| VEGF pg/ml | 116.92 [98.07-191.71] | 194.11 [87.66-246.85] | 0.37 |
| TGF-β pg/ml | 13.013.84 [10.450.04-22.082.96] | 11386.62 [6.425.03-20.903.94] | 0.48 |

IL: interleukin; HGF: hepatocyte growth factor,; IGF: insulin-like growth factor; TGF-β: transforming growth factor β; VEGF: vascular endothelial growth factor.
